# Editorial: Identifying the isoform-specific roles of RAS paralogs in health and disease

**DOI:** 10.3389/fcell.2023.1199356

**Published:** 2023-04-13

**Authors:** Saeideh Nakhaei-Rad, Anna Fejtova

**Affiliations:** ^1^ Stem Cell Biology and Regenerative Medicine Research Group, Institute of Biotechnology, Ferdowsi University of Mashhad, Mashhad, Iran; ^2^ Institute of Biochemistry and Molecular Biology II, Medical Faculty and University Hospital Düsseldorf, Heinrich Heine University Düsseldorf, Düsseldorf, Germany; ^3^ Department of Psychiatry and Psychotherapy, Universitätsklinikum Erlangen, Friedrich-Alexander-Universität Erlangen-Nürnberg, Erlangen, Germany

**Keywords:** RAS, effector, isoform, paralog, cancer, rasopathy

The RAS family of small GTPases is categorized into eight groups of paralogs: RAS, RAL, RRAS, RIT, RAP, RHEB, RASD, ERAS, and DIRAS. Although the prototypes of the RAS family, KRAS, NRAS, and HRAS, are well-known for their role in human carcinogenesis, RAS proteins also play fundamental roles in normal human development, by regulating a wide array of cellular processes, including survival, growth, adhesion, migration, differentiation and fate decision (Nakhaei-Rad et al., 2018). The dysregulation of the RAS signaling pathway leads to cancer as well as to developmental disorders, named RASopathies, connected with cardiac, skin, neuronal, and metabolic phenotypes.

At first glance, the RAS family with 26 isoforms and paralogs that harbor the conserved motifs and regions was tempting to have functional redundancy. However, new evidence indicates that each RAS family member harbors also number of specific features that make them to some degree unique in regulation, subcellular localization, effector selection, signaling strength, dynamics, timing, and networking (Nussinov et al., 2018; Hood et al., 2019; Nair et al., 2021; Pudewell et al., 2021; Simanshu and Morrison, 2022). Despite more than four decades of research on RAS, our understanding of the functional differences between the RAS isoforms and paralogs is very limited.

More dimensionality in RAS functions emerges from their tightly regulated transcription and translation by several molecular mechanisms during the cell cycle phases, developmental stages, tissue specifications, and in response to the various stimuli, stressors, and pathogenic conditions (Newlaczyl et al., 2017; Nussinov et al., 2021; Saliani et al., 2022; Hood et al., 2023). This topic includes one research and review articles that discuss the splice variants of small GTPases. Das et al. perform a comprehensive analysis of the small GTPase SpliceOme in various human tissues and highlight the impacts of environmental factors, age, sex, and RNA sequencing strategies on splicing dynamics. Philips and Nuevo-Tapioles describe and dissect in great detail the functional differences among two prominent KRAS splice variants, KRAS4A, and KRAS4B, in cancer development, metabolism, and progression.

The RAS signaling propagation and functional outcomes rely on their association and modulation of the diverse spectrum of downstream effector proteins. Of note, the functional specificity of the RAS isoforms and paralogs comes in part from their affinity and access to the specific effector proteins. The RAS-effector association is mainly conducted through the RAS-binding (RBD) or RAS association (RA) domains of the effectors. RAF and PI3 kinases, due to their impacts on the oncogenic functions of RAS, are well-investigated RAS effectors (Nakhaeizadeh et al., 2016; Rezaei Adariani et al., 2018). However, there are more than 57 proteins that contain the RA domain and RBD that also need to be further investigated as putative RAS effectors (Rezaei Adariani et al., 2021). This issue is addressed in the topic. Pudewell et al. focus on detailed aspects of the stress-activated MAP kinase-interacting protein 1 (SIN1) interaction mode with RAS paralogs and various types of membrane phosphoinositides. SIN1, a critical subunit of the mTOR complex 2, is a newly discovered RAS effector that harbors the RBD, and a pleckstrin homology (PH) domain. They show that among RAS isoforms, SIN1-RBD binds more tightly to classical RAS family members and introduces new binding partners. The authors’ findings suggest that RAS interaction influences the membrane recruitment of SIN1.

To date, the majority of the available information about the RAS isoforms is obtained from studying human cancers. However, we need to admit that a wide spectrum of variable factors in cancer cells such as genome instability, chromosomal abnormalities, gene amplifications, presence of other mutations, and heterogeneous nature, will affect these findings. The germline point mutations of the RAS genes and their signaling components cause a group of syndromes including Cardio-Facio-Cutaneous (CFC), Costello (CS), Legius (LS), Neurofibromatosis type 1 (NF1), Noonan (NS), and Noonan-like (NS with Multiple Lentigines, NSML) that are collectively known as RASopathies (Caye et al., 2015; Altmüller et al. 2017; Motta et al., 2020; Zenker, 2022). RASopathy patients carry a point mutation in one of the RAS signaling components or regulators leading to aberrant RAS signaling in all cells and tissues of the body, unravelling the tissue-specific function of specific RAS isoforms and RAS-MAPK signaling in general. Nandi et al. investigate the impacts of the HRAS^G12V^ mutation on bone loss and osteoclast differentiation and function in the mouse model of CS. They introduce the HRAS isoform as a regulator of bone homeostasis and show that its deregulation results in osteoporosis.

The development of advanced molecular approaches, including high-throughput molecular technologies, super-resolution microscopy techniques, computational models, genome editing, and single-cell analysis would have a great impact on improving our understanding of the cell-type and context-dependent functions of RAS isoform and paralogs in normal and pathological conditions. We hope that this Research Topic inspire researchers to more systematically and comprehensively investigate the underscored aspects of the individual RAS proteins and their signaling networks.
